# Chronology of embryonic and gonadal development in the Reeves’ turtle, *Mauremys reevesii*

**DOI:** 10.1038/s41598-022-15515-w

**Published:** 2022-07-08

**Authors:** Hiroshi Akashi, Manami Kubota, Hibiki Yamamoto, Kaori Miyaoku, Genki Yamagishi, Shinichi Miyagawa

**Affiliations:** 1 Department of Biological Science and Technology, Faculty of Advanced Engineering, Tokyo University of Science, 6-3-1 Niijuku, Katsushika-ku, Tokyo, 125-8585 Japan; 2 Research Institute for Science and Technology, Tokyo University of Science, Tokyo, 125-8585 Japan

**Keywords:** Developmental biology, Zoology

## Abstract

Temperature-dependent sex determination (TSD) is a mechanism in which environmental temperature, rather than innate zygotic genotype, determines the fate of sexual differentiation during embryonic development. Reeves’ turtle (also known as the Chinese three-keeled pond turtle, *Mauremys reevesii*) exhibits TSD and is the only species whose genome has been determined in Geoemydidae to date. Thus, *M. reevesii* occupy phylogenetically important position for the study of TSD and can be compared to other TSD species to elucidate the underlying molecular mechanism of this process. Nevertheless, neither embryogenesis nor gonadogenesis has been described in this species. Therefore, herein, we investigated the chronology of normal embryonic development and gonadal structures in *M. reevesii* under both female- and male-producing incubation temperatures (FPT 31 °C or MPT 26 °C, respectively). External morphology remains indistinct between the two temperature regimes throughout the studied embryonic stages. However, the gonadal ridges present on the mesonephros at stage 16 develop and sexually differentiate at FPT and MPT. Ovarian and testicular structures begin to develop at stages 18–19 at FPT and stages 20–21 at MPT, respectively, and thus, the sexual differentiation of gonadal structures began earlier in the embryos at FPT than at MPT. Our results suggest that temperature sensitive period, at which the gonadal structures remain sexually undifferentiated, spans from stage 16 (or earlier) to stages 18–19 at FPT and to stages 20–21 at MPT. Understanding the temperature-dependent differentiation in gonadal structures during embryonic development is a prerequisite for investigating molecular basis underlying TSD. Thus, the result of the present study will facilitate further developmental studies on TSD in *M. reevesii*.

## Introduction

Sexually dimorphic phenotypes of vertebrates primarily emerge as gonadal sex differentiation during embryonic development. In vertebrates, genetic sex determination is a mechanism of differentiating gonadal sex from a bipotent or undifferentiated state, relying on the dimorphic expression of sex-determining genes usually derived from sexually heteromorphic chromosomes^1–3^. In contrast, alternative sex determination mechanisms are governed by environmental cues, among which temperature is one of the most prevalent in reptile sex determination. Temperature-dependent sex determination (TSD) is documented for most turtles, some lizards, all crocodilians, and tuataras, but not snakes^4–6^.

The fate of sexual differentiation in TSD species strongly depends on the environmental temperatures that individuals experience during a critical developmental period, the temperature sensitive period (TSP)^7–9^. TSP has historically been deduced from experiments that shift the egg incubation temperatures during the embryonic development and analyze the resulting sex of the individuals. Interestingly, TSP in turtles and alligator is closely associated with the sexual differentiation of gonadal structures^8,9^. In vertebrates, the coelomic epithelium thickens and develops into the genital ridges on the mesonephros at early embryonic stages^10^. The red-eared slider turtle *Trachemys scripta* shows this general gonadal development pattern, and the beginning of its TSP corresponds to the early stage at which the genital ridges develop, i.e., stage 16 (or earlier)^8^. The TSP in *T. scripta* then lasts until the sexually bipotential gonads acquire the ovarian or testicular structures, corresponding to stages 18–19 or 20–21 when incubated under 31 °C or 26 °C, respectively^8^. After the sexually dimorphic gonads develop, the sex is no longer reversible regardless of the changes in incubation temperature.

Studies on the molecular basis of TSD often investigate the expression of gonadal genes throughout embryonic stages, particularly focusing on the TSP. Recent advents in sequencing techniques have revealed genes presumably involved in gonadal sex differentiation or sexually distinct transcription in TSD species^11–16^, which were further investigated in *Alligator mississippiensis* and *T. scripta* embryos using techniques, such as pharmacological treatments^12^ and genetic manipulations via viral infections^17,18^. These manipulations were used to evaluate whether the sex expected from a female- or male-producing incubation temperature (FPT or MPT, respectively) could be reversed, assuming that gonadal sex was structurally undifferentiated at the time of these manipulations. Understanding the structural differentiation of gonads throughout embryonic stages is a prerequisite for these studies; this information is available for *A. mississippiensis* and *T. scripta*, which makes these species the best models for TSD studies.

Our main objective in the present study is to describe the differentiation of gonadal structures throughout the normal embryonic stages of Reeves’turtle (also known as the Chinese three-keeled pond turtle, *Mauremys reevesii*), a freshwater species that prevalently inhabits East Asia^19^. *Mauremys reevesii* is the only species whose genome has been determined in Geoemydidae to date^20^, thereby making it phylogenetically important for future comparative studies within and/or between TSD and genotypic sex determination (GSD) turtle species. While *T. scripta* is an excellent model for the study of TSD, the International Union for Conservation of Nature (IUCN) listed *T. scripta* as one of the worst invasive species outside their native distribution^21^. Due to the ecological status in *T. scripta*, *M. reevesii* is a good alternative study model particularly in Asia. Although wild populations of *M. reevesii* are reportedly under threat in China due to habitat change and human exploitation, cultured individuals are commercially available in China^22^ and Japan. Du et al.^22^ reported the TSD of *M. reevesii*, with a heavily female- and male-biased sex ratio of hatchlings at incubation temperatures above 30 °C and below 26 °C, respectively. This pattern is TSD Ia (i.e., the proportion of females increase with incubation temperature), as in *T. scripta*^5^. Thus, *M. reevesii* can be compared to *T. scripta* to better understand the temperature dependency of sex determination. As the normal embryonic development and the differentiation of gonadal structures in *M. reevesii* have not yet been described, the present study investigates the following in *M. reevesii*: (1) normal embryonic stages, (2) developmental rate under both FPT and MPT, and (3) differentiation of gonadal structures at each embryonic stage. Our findings will provide a basis for effective sampling of embryos and the design of *in ovo* experiments using *M. reevesii*.

## Results

**Figure 1:**
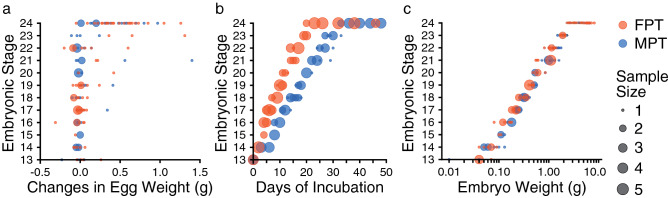
Characteristics of stages 13–24 in *Mauremys reevesii* as represented by egg weight, incubation days, and embryo weight. (**a**) Egg weight for each stage are shown as a difference that the weight on the day of dissection is subtracted by the weight on the day of arrival. (**b**) Days of incubation from stage 13 required to reach each stage are shown. (**c**) Embryo weight at stages 13–24 are shown without the extraembryonic membranes. FPT and MPT denote female- and male-producing temperatures (31 °C and 26 °C), respectively. The size of dots corresponds to the sample sizes.

**Figure 2:**
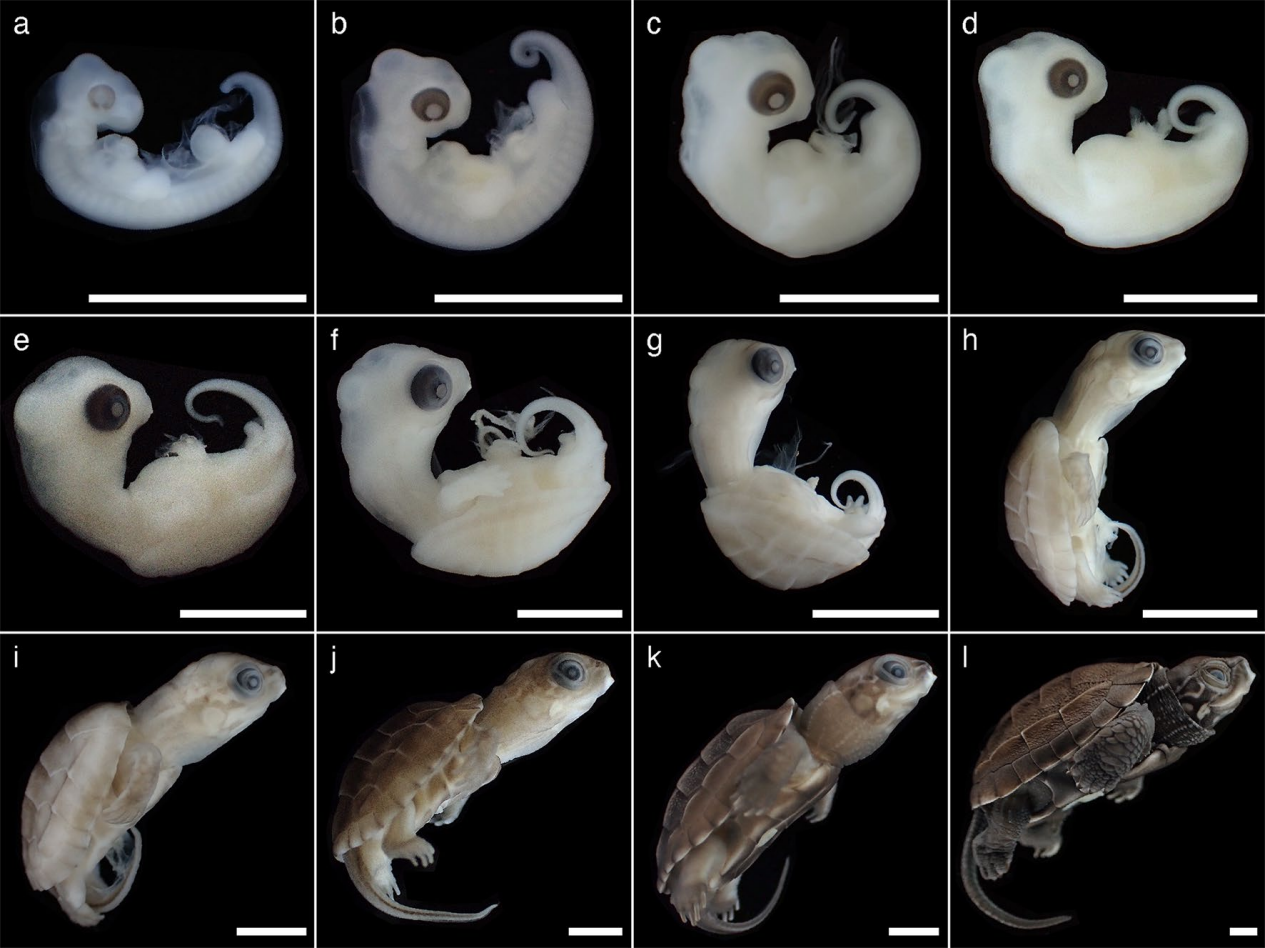
Stages 13–24 of *Mauremys reevesii* in lateral view. (**a**) Stage 13. (**b**) Stage 14. (**c**) Stage 15. (**d**) Stage 16. (**e**) Stage 17. (**f**) Stage 18. (**g**) Stage 19. (**h**) Stage 20. (**i**) Stage 21. (**j**) Stage 22. (**k**) Stage 23. (**l**) Stage 24. The scale bar is 5 mm.

**Figure 3:**
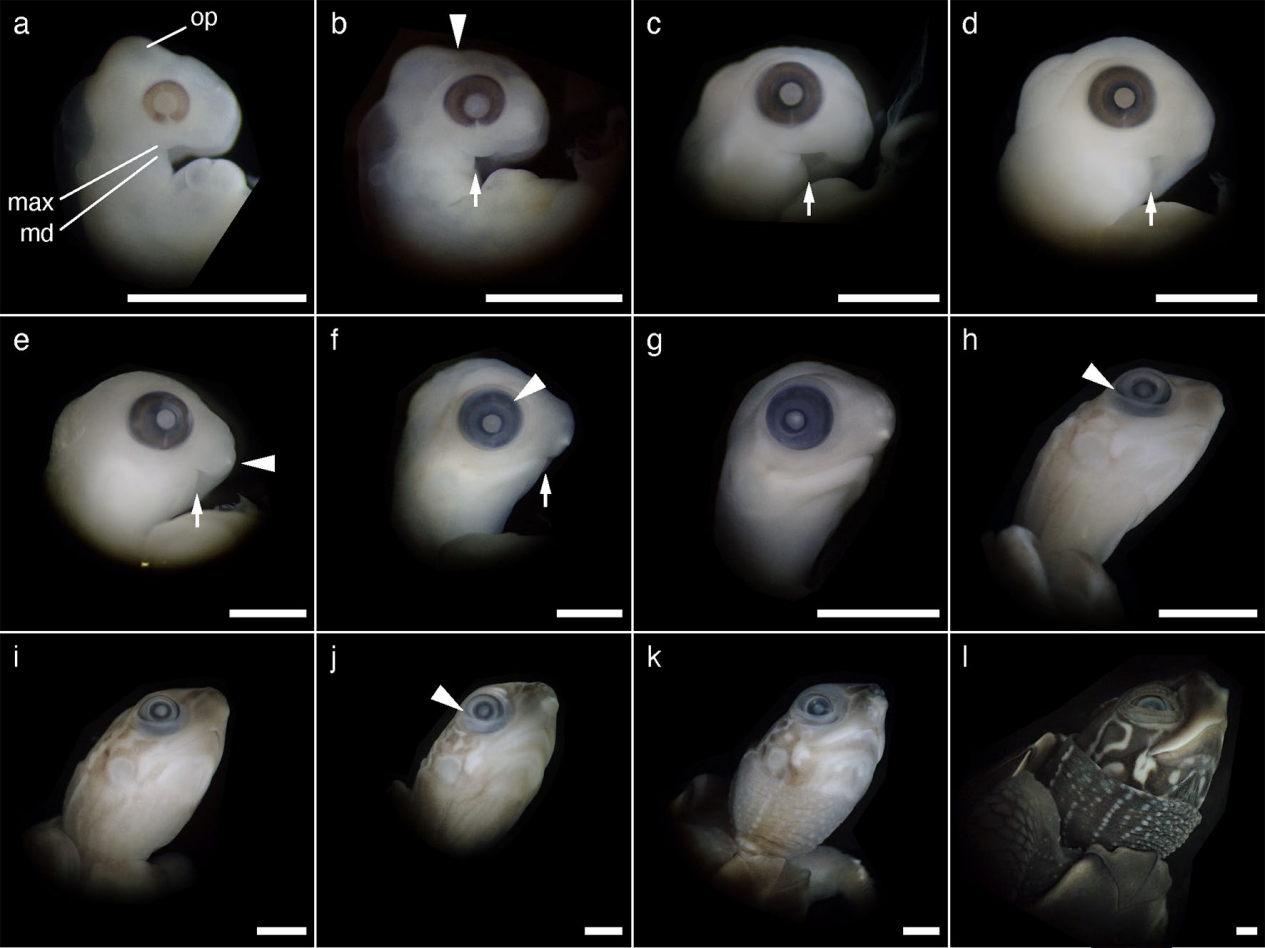
Formations of eye, mandible, and the surrounding structures at stages 13–24 of *Mauremys reevesii*. The arrows indicate the anterior end of maxillary process. (**a**) Stage 13. (**b**) Stage 14. The triangle points to the anterior end of the occipital protuberance. (**c**) Stage 15. (**d**) Stage 16. (**e**) Stage 17. The triangle points to the caruncle. (**f**) Stage 18. The triangle points to one of the scleral papillae. (**g**) Stage 19. (**h**) Stage 20. The triangle points to the lower eyelid reaching the scleral papillae. (**i**) Stage 21. (**j**) Stage 22. The triangle points to the lower eyelid reaching outer margin of the iris. (**k**) Stage 23. (**l**) Stage 24. op, the occipital protuberance; max, maxillary process; md, mandibular process. The scale bar is 2.5 mm.

**Figure 4:**
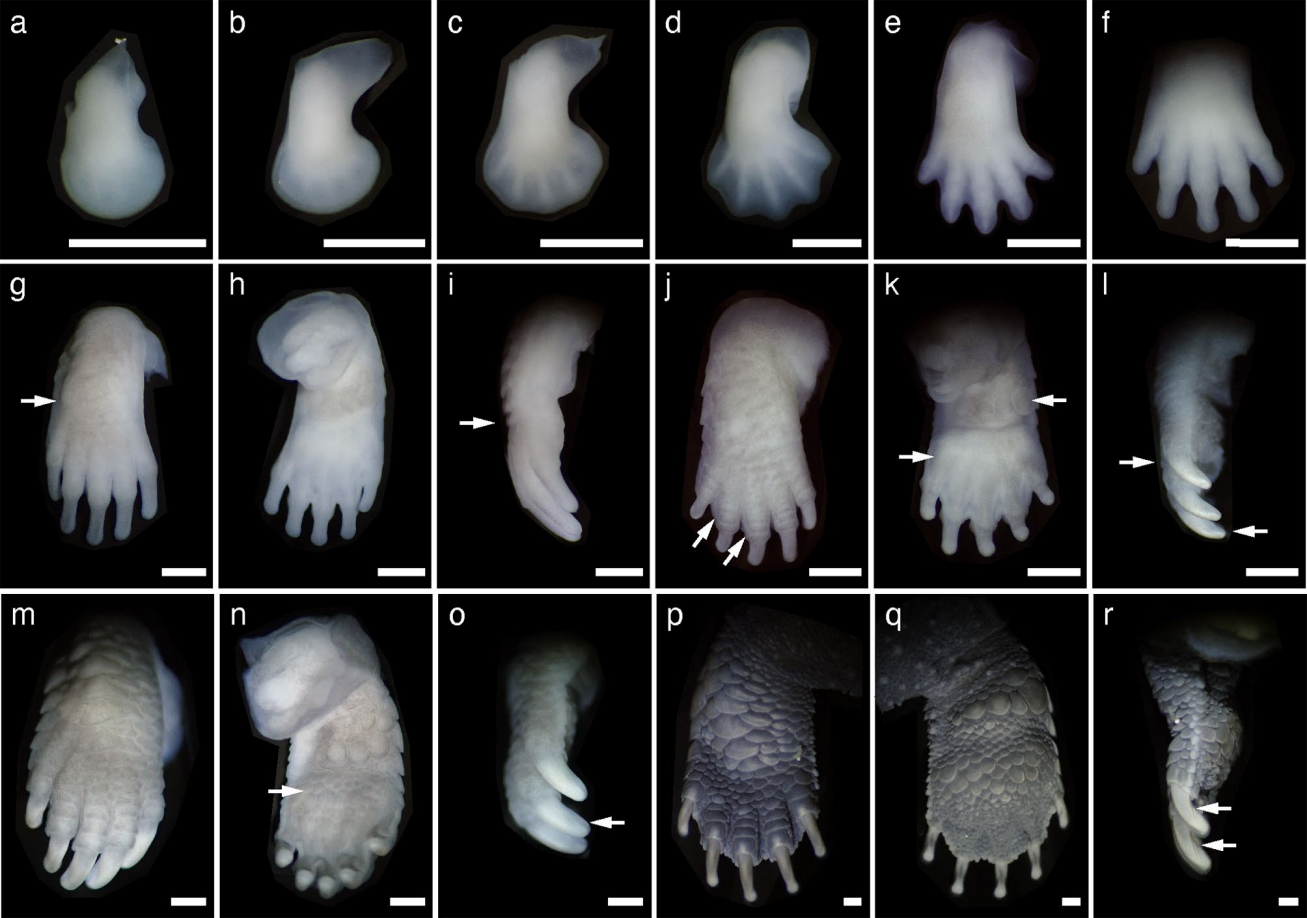
The right forelimb of *Mauremys reevesii* at stages 15–24, shown in dorsal (**a**–**g**,**j**,**m**,**p**), ventral (**h**,**k**,**n**,**q**), and lateral view (**i**,**l**,**o**,**r**). (**a**) Stage 15. (**b**) Stage 16. (**c**) Stage 17. (**d**) Stage 18. (**e**) Stage 19. (**f**) Stage 20. (**g**) Stage 21. The arrow indicates that the scales on the dorsal forelimb do not reach the proximal border of the webbing. (**h**) Stage 21. (**i**) Stage 21. The arrow indicates the scales on the dorsal forelimb in lateral view for clarity. (**j**) Stage 22. Both arrows indicate the scales extend to the distal end of the digits. (**k**) Stage 22. The upper arrow indicates four large circular scales aligned in line. The lower arrow indicates the slight formation of small circular scales on palm. (**l**) Stage 22. The upper arrow indicates the scales on the dorsal forelimb in lateral view for clarity. The lower arrow indicates the tip of the ungual phalanx visible in a translucent sheath. (**m**) Stage 23. (**n**) Stage 23. The arrow indicates the palmar surface of the forelimb is fully covered by small circular scales. (**o**) Stage 23. The arrow indicates the ungual phalanx evident in a translucent sheath. (**p,q**) Stage 24. (***r***) Stage 24. The translucent sheath on the ungual phalanx recedes toward its distal end. The scale bar is 1 mm.

**Figure 5:**
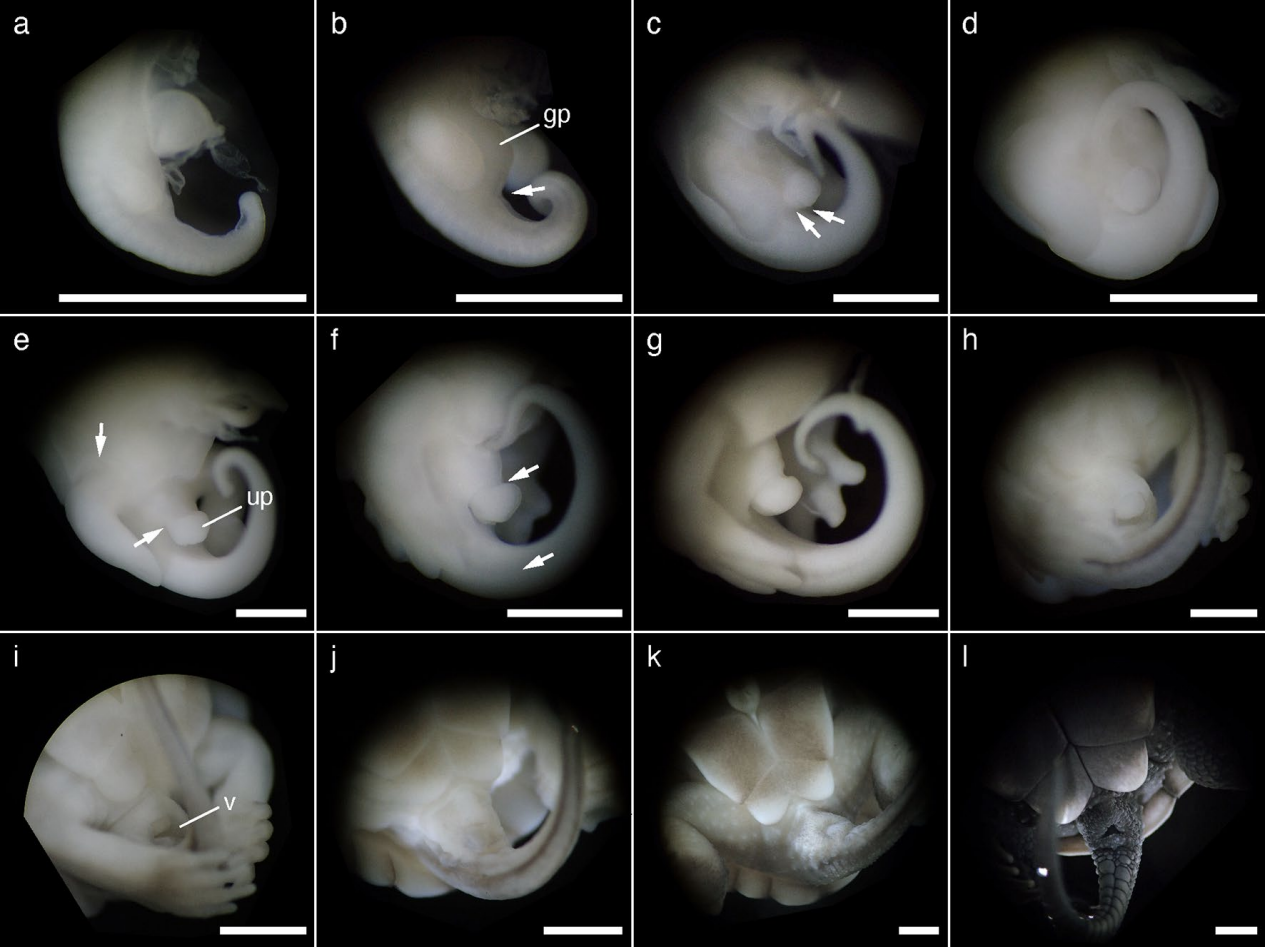
Formation of cloaca and its surrounding structures at stages 13–24 of *Mauremys reevesii*. (**a**) Stage 13. Genital protuberance is still absent. (**b**) Stage 14. The arrow indicates a slight crease. (**c**) Stage 15. The left arrow indicates a crease between genital protuberance and tail, and the right arrow indicates a crease distinguishing urogenital papilla. (**d**) Stage 16. (**e**) Stage 17. The upper arrow indicates the posterior border of the plastron. The lower arrow indicates a crease. (**f**) Stage 18. The upper arrow indicates a fold surrounding the urogenital papilla. The lower arrow indicates a line of pigmentation. (**g**) Stage 19. (**h**) Stage 20. (**i**) Stage 21. (**j**) Stage 22. (**k**) Stage 23. (**l**) Stage 24. gp, genital protuberance; up, urogenital papilla; v, vent. The scale bar is 2.5 mm.

**Figure 6:**
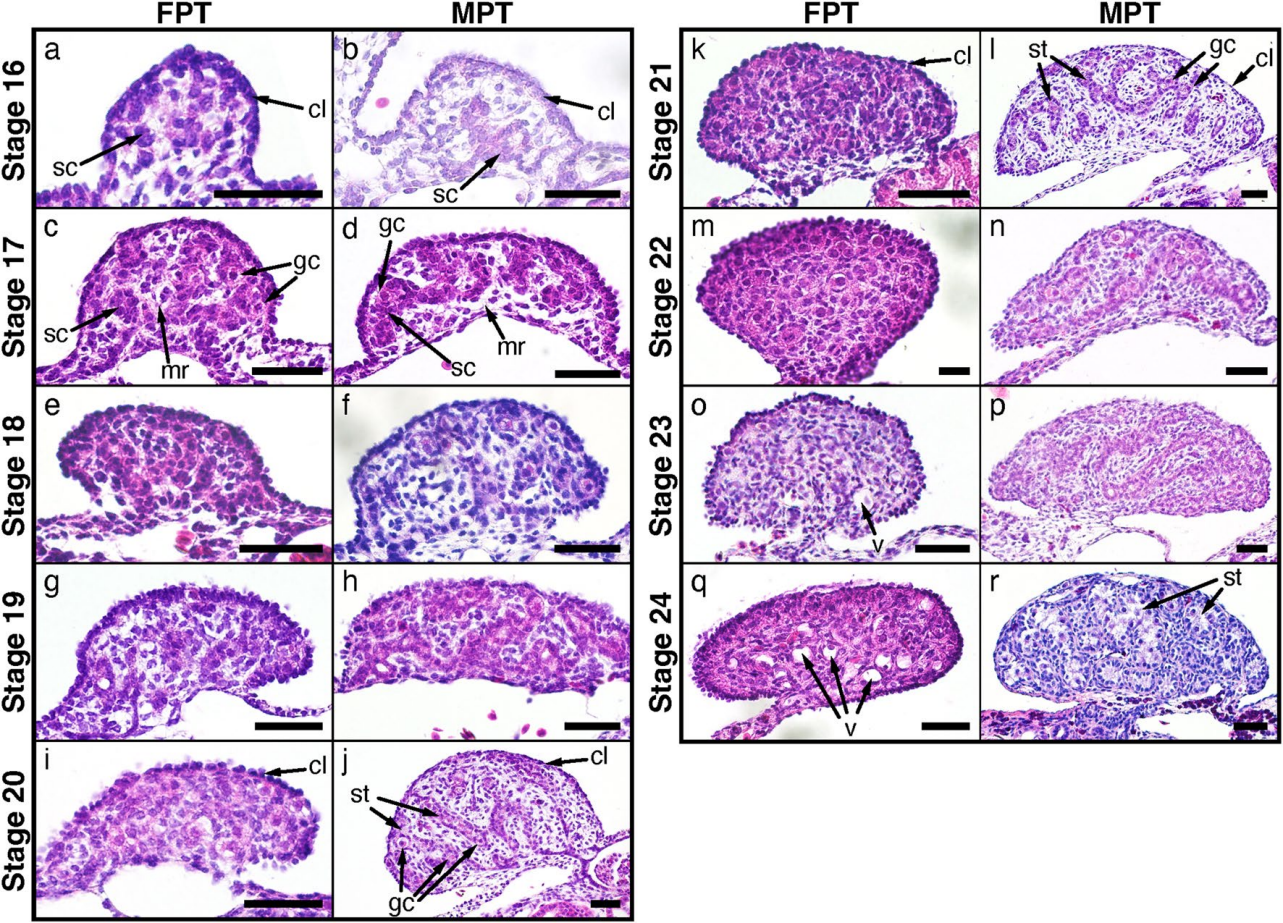
Gonadal development of *Mauremys reevesii* during stages 16–24 at female- and male-producing temperatures (FPT 31 °C and MPT 26 °C, respectively). (**a**) Stage 16, FPT (n = 1). (**b**) Stage 16, MPT (n = 1). (**c**) Stage 17, FPT (n = 4). (**d**) Stage 17, MPT (n = 3). (**e**) Stage 18, FPT (n = 3). (**f**) Stage 18, MPT (n = 3). (**g**) Stage 19, FPT (n = 5). (**h**) Stage 19, MPT (n = 3). (**i**) Stage 20, FPT (n = 2). (**j**) Stage 20, MPT (n = 4). (**k**) Stage 21, FPT (n = 3). (**l**) Stage 21, MPT (n = 4). (**m**) Stage 22, FPT (n = 7). (**n**) Stage 22, MPT (n = 5). (**o**) Stage 23, FPT (n = 3). (**p**) Stage 23, MPT (n = 3). (**q**) Stage 24, FPT (n = 4). (**r**) Stage 24, MPT (n = 4). sc, sex cord; cl, cortical layer; mr, medullary region; gc, germ cell; st, seminiferous tubule; v, vacuolated area. The scale bar is 50 µm.

We described the external morphology of *M. reevesii* embryos at stages 13–24 and their gonadal structure at stages 16–24. Eye pigmentation was first recognized by candling at stage 13; thus, our analysis began at stage 13. The number of embryos analyzed in this study along with the changes in the egg weight, egg incubation days, embryo weight, incubation temperatures, and key characteristics used for staging *M. reevesii* are summarized in Fig. 1, Table 1 and Supplementary Table S1. The results of the statistical analyses are summarized in Supplementary Table S2. The changes in egg weight were not correlated with the embryonic stage (*P*-value = 0.06); however, we found significant positive correlations between the embryonic stage and both the egg incubation days and the embryo weight (*P*-value < 2.0 × 10^–16^ and *P*-value < 2.0 × 10^–16^, respectively). The result also showed a significant interaction between the incubation temperatures and the egg incubation days (*P*-value < 2.0 × 10^–16^). Neither the changes in egg weight nor the embryo weight showed a significant interaction with the incubation temperatures (*P*-value = 0.50 and *P*-value = 0.17, respectively). According to the Pearson's correlation coefficient (*r*), the egg incubation days at FPT (*r* = 0.98, *P*-value < 2.2 × 10^–16^) and MPT (*r* = 0.99, *P*-value < 2.2 × 10^–16^), and the embryo weight (*r* = 0.98, *P*-value < 2.2 × 10^–16^) showed strong correlations with the embryonic stage while the correlation between the changes in egg weight and the embryonic stage was weak with *r* to be 0.32 (*P*-value = 6.8 × 10^–4^). No sexual dimorphic features were observed with respect of the external morphology during the embryonic development at either FPT (31 °C) or MPT (26 °C). The representative individual of each stage is shown in Fig. 2 in lateral view, and Supplementary Figures S1 and S2 in dorsal and ventral view, respectively; mandible formations are shown in Fig. 3. The representative digit formations of selected stages are shown in Fig. 4, while those of studied stages are shown in Supplementary Figures S3 and S4 in dorsal and ventral view, respectively. Formations of genital protuberances, urogenital papillae, and vent are shown in Fig. 5. Histology sections of representative gonads for both FPT and MPT are shown in Fig. 6.

**Table 1:** The total sample size, days required to reach each stage from stage 13 (mean ± standard deviation, SD) at FPT or MPT, embryo weight at the time of dissection (mean ± SD), and the key characteristics used in this study for staging *Mauremys reevesii*. FPT and MPT denote female- and male-producing incubation temperatures (31 °C and 26 °C, respectively).

Stage	Sample size		Days to stage		Embryo (g)		Key characteristics
	FPT	MPT	FPT	MPT	FPT	MPT	
13	4	4	0	0	0.05 ± 0.02	0.03 ± 0.02	Pigmented over retina
14	6	8	1.7 ± 0.8	4.5 ± 1.6	0.06 ± 0.02	0.08 ± 0.03	Pigmentation reaches pupil
15	2	4	4	8	0.11 ± 0.01	0.11 ± 0.01	Lateral margin of carapacial ridge is evident
16	7	6	4.4 ± 0.5	9.3 ± 1.0	0.14 ± 0.03	0.17 ± 0.01	Anterior end of carapacial ridge is evident
17	11	7	6 ± 0.8	12 ± 1.0	0.22 ± 0.03	0.25 ± 0.02	Posterior margin of plastron is evident
18	8	13	8.4 ± 0.9	15.4 ± 1.6	0.31 ± 0.05	0.34 ± 0.04	Digits II–IV protrude, and the thickness at the web is greater than the protrusion
19	9	6	10.2 ± 0.7	17.3 ± 0.8	0.43 ± 0.03	0.53 ± 0.03	Digits II–IV protrude as much as or more than the thickness at the web
20	5	5	11.8 ± 0.8	20.4 ± 0.9	0.62 ± 0.14	0.60 ± 0.15	Digits II–IV protrude approximately twice their thickness at the web
21	8	9	15 ± 0.9	24 ± 2.2	0.88 ± 0.28	1.07 ± 0.25	The scales on the dorsal forelimb are slightly visible, but do not reach the proximal border of the webbing
22	10	8	15.8 ± 1.4	26.4 ± 1.8	1.13 ± 0.18	1.42 ± 0.20	The scales on the dorsal forelimb extend to the ranges from the proximal border of the webbing to the distal region of the digits
23	6	8	19.5 ± 0.5	29.3 ± 2.0	1.92 ± 0.14	2.00 ± 0.25	The palmar surface of the forelimb is fully covered by small circular scales
24	25	19	30.9 ± 8.2	41 ± 5.2	4.61 ± 1.70	4.92 ± 1.36	The translucent sheath on the ungual phalanx recede toward its distal end

### Embryonic development

#### Stage 13

In the lateral view, the anterior edge of the occipital protuberance extends anteriorly beyond the posterior edge of the eye (Figs. 2a, 3a). In the same view, the maxillary process encroaches anteriorly beyond the optic fissure (Figs. 2a, 3a). Pigmentation of the eye is dense, but the pupil and its margin are unpigmented (Figs. 2a, 3a). The optic fissure appears as an unpigmented “triangular” slit at the ventral region of the eye (Fig. 3a). The anterior end of the mandible lies anteriorly beyond the level of the posterior edge of the eye (Fig. 3a). The forelimb buds are longer than wide (Supplementary Fig. S3a, S4a).

#### Stage 14

In the lateral view, the occipital protuberance recedes posteriorly as its anterior edge is near the level of the posterior edge of the eye (Figs. 2b, 3b). The area between the maxillary and lateral nasal processes—where the external nares are later formed—is weakly marked by shallow creases. The pigmentation of the iris reaches the margin of the unpigmented white pupil (Figs. 2b, 3b). The forelimb bends in caudal orientation (Fig. 2b). The body is more flexed ventrally and the tail is longer and more curled than at stage 13 (Fig. 2b). The genital protuberance is evident, with a slight crease at its posterior margin (Fig. 5b).

#### Stage 15

The anterior end of the mandible extends to the level of the center of the eye (Fig. 3c). The carapacial ridge becomes visible in lateral view but its anterior edge remains absent and smooth (Fig. 2c). The digital plate is well formed (Fig. 4a). The posterior border of the genital protuberance is evident by a crease between the genital protuberance and the tail (Fig. 5c). The urogenital papilla is slightly distinguished from the entire genital protuberance by a shallow crease (Fig. 5c). The dorsal surface is slightly pigmented from the anterior edge of the carapace to the nostril and at the base of the tail.

#### Stage 16

The optic fissure is evident but becomes narrower, from a “triangular” to “teardrop” shape (Fig. 3d). The anterior end of the mandible extends beyond the level of the center of the eye (Fig. 3d). An incipient tympanum is barely visible in lateral view. The digital plate has a smooth periphery (Fig. 4b). The carapacial ridge becomes thicker and its anterior edge is distinct in the lateral view (Fig. 2d). The pigmentation becomes denser on the dorsal surface of the neck and head regions and at the base of the tail.

#### Stage 17

The optic fissure is visible but becomes a narrow slit (Fig. 3e). The caruncle is visible as a small white mark on the rostral tip of the upper jaw (Fig. 3e). The mandible extends anteriorly, the ending of which is between the level of the anterior end of the eye and that of the nostril in the lateral view (Fig. 3e). Each of the five digits can be distinguished by a ridge as the digital plate is slightly serrated (Fig. 4c). The posterior border of the plastron is evident (Fig. 5e). A crease on the posterior border of the genital protuberance extends more laterally (Fig. 5e). The urogenital papilla becomes larger and protrudes as it occupies a large proportion of the genital protuberance (Fig. 5e). Ribs can be slightly seen through the carapace in dorsal view (Supplementary Fig. S1e).

#### Stage 18

The optical fissure disappears at this stage, and scleral papillae are evident (Fig. 3f). The caruncle is more evident (Fig. 3f). The mandible and the upper jaw make an almost complete closure as the mandible extends anteriorly beyond the level of the anterior end of the eyes in the lateral view (Fig. 3f). Digits II–IV protrude along the periphery of the digital plate but the thickness at the web is greater than the protrusion (Fig. 4d). Carapace pigmentation begins at this stage 18, such that the dorsolateral margins of the carapace are slightly pigmented, exposing the marginal scutes with faint borders (Supplementary Fig. S1f). The plastron remains unpigmented, but it weakly shows the borders of the plastral scutes. The lateral sides of the tail are slightly pigmented, forming lines of pigments (Fig. 5f). A fold is evident surrounding the urogenital papilla (Fig. 5f).

#### Stage 19

The pigmentation on the head region becomes denser and the external nares are emphasized by the round, unpigmented areas. The lower eyelid is slightly formed. Scleral papillae are distinct (Fig. 3g). The mandible and the upper jaw make a complete closure (Fig. 3g). Digits II–IV protrude as much as or more than the thickness at the web along the periphery of the digital plate (Fig. 4e). The carapace becomes more pigmented, evidencing the borders of all scutes (Supplementary Fig. S1g). The three keels on the carapace are barely visible; one runs along the midline of the vertebral scutes and the other two run through the costal scutes (Supplementary Fig. S1g).

#### Stage 20

The lower eyelid reaches the scleral papillae (Fig. 3h). The protrusion of digits II–IV proceeds and reaches approximately twice their thickness at the web (Fig. 4f). The digits are weakly pigmented (Fig. 4f). Rows of cutaneous papillae are slightly visible on the dorsal surface of the neck. The entire carapace is pigmented, but the vertebral scutes are more pigmented than the other scutes (Supplementary Fig. S1h). The cutaneous papillae on the vertebral scutes are barely visible in some specimens. The urogenital papilla begins to withdraw into but remains prolapsed from the vent (Fig. 5h).

#### Stage 21

The scleral papillae disappear or become unclear at this stage (Fig. 3i). The entire body becomes more evidently pigmented (Fig. 2i, Supplementary Figs. S1i, S2i). The pigmentation of the digits becomes denser at the thinner distal ends than the thicker proximal regions on most of specimens, vaguely indicating the border of digits and claws (Fig. 4g). The claws are homogeneously opaque and white-colored with evident pigmentation (Fig. 4i). The scales are slightly visible on the dorsal forelimb, but do not reach the proximal border of the webbing (Fig. 4g,i). The cutaneous papillae on the vertebral scutes are evident. The urogenital papilla withdraws into the vent (Fig. 5i).

#### Stage 22

The iris becomes distinct and the lower eyelid reaches its outer margin (Fig. 3j). The ungual phalanx enclosed in a translucent sheath is clearly visible including its tip (Fig. 4l). Pigmentation becomes heavier on the dorsal forelimb (Fig. 4j). The scales on the dorsal forelimb extend distally to the ranges of the proximal border of the webbing to the distal region of the digits (Fig. 4j,l). The palmar surface of the forelimb is slightly covered by small circular scales (Fig. 4k), and in some specimens, these scales overlap adjacent scales. Four large circular scales are more evident and aligned along the anteroposterior axis of the palmar surface (Fig. 4k). Each of these four scales slightly overlaps or projects over the surrounding palmar surface. The carapace becomes darker so that the presence of cutaneous papillae on the entire carapace is emphasized. The intestinal loop is withdrawn into the body (Supplementary Fig. S2j).

#### Stage 23

The ungual phalanx is evident in a translucent sheath (Fig. 4o). The palmar surface of the forelimb is fully covered by small circular scales (Fig. 4n), and their overlap to adjacent scales is evident. The density of the cutaneous papillae increased on the vertebral and pleural scutes. The plastron is more pigmented so that the whitish regions are less than the darker pigmented regions (Supplementary Fig. S2k).

#### Stage 24

The embryos resemble the hatchlings. The translucent sheath on the ungual phalanx recedes toward its distal end, exposing the ventral surface of the ungual phalanx (Fig. 4r). The individual scales on the forelimb, hindlimb, and tail are evident and overlap one another. The pigmentation becomes darker throughout the body; the pigmentation of the carapace is dark brown in color but that of the plastron and skin is blackish brown. The color contrast increases on the head, exposing a discernible pattern on the lateral and ventral surface of the head (Fig. 3l).

### Gonadal development

At stage 16 of both FPT and MPT, the gonadal ridges with inner medulla consisting of sex cords and outer 1–2 cell cortical layer are present on the mesonephros (Fig. 6a,b). At stage 17 of both FPT and MPT, the germ cells with large spherical nuclei and the medullary sex cords become more evident (Fig. 6c,d). At stage 18, the sex cords begin to degenerate in some specimens at FPT (Fig. 6e). By stage 20, the sex cords become disorganized, and the medullary region shows the homogeneous structure at FPT (Fig. 6i), while at MPT, the sex cords become more distinct, developing into the seminiferous tubules (Fig. 6j). As the seminiferous tubules develop at stages 20 and 21, the germ cells are enclosed within the cords at MPT (Fig. 6j,l). During stages 20 and 21, the cortex becomes enlarged and thickened at FPT (Fig. 6i,k); conversely, the cortex becomes a thin one-cell layer at MPT (Fig. 6j,l). From stage 21, the tissue area connecting gonads and the mesonephros begins to decrease, with the gonads becoming merely connected by a narrow stalk of the connective tissues by stage 24 (Fig. 6q,r). At stages 23 and 24, the distinction between cortex and medullary region is more evident and the medullary region becomes vacuolated at FPT (Fig. 6o,q). At stage 24, the seminiferous tubules are developed at MPT (Fig. 6r).

All specimens after stage 21 show sexually distinct gonadal structures, and develop ovarian or testicular structures as expected from the incubation temperatures of FPT or MPT, respectively. No specimen shows gonadal sex reversed from the expected sex at FPT or MPT. The macro images of the gonads at stage 24, or pre-hatching stage, are shown in Supplementary Figure S5.

## Discussion

The sex of TSD species is determined during embryonic development, particularly during the TSP, depending on thermal environmental conditions^5^. Sex determination is primarily recognized by the differentiation of gonadal structures toward testicular or ovarian or testicular developmental pathways. While *M. reevesii* has been reported to be a TSD species, the chronology of the sexual differentiation of its gonadal structures has not been investigated. In the present study, we analyzed the gonadal development of *M. reevesii* under both FPT and MPT according to the chronology of its external embryonic morphogenesis.

The embryonic development of *M. reevesii* regarding its external morphology was analyzed following criteria developed for *C. serpentina*^23^ and with a particular reference to the testudinoid species, *T. scripta*^24^ and *Mauremys japonica*^25^. Several features of *M. reevesii* were distinct from those of both or either of the latter two species. The claw development is a key to discerning later stages in *T. scripta* and particularly in *M. japonica*, but we recognized the characteristics of the claw development at distinct times between *M. reevesii* and *T. scripta*/*M. japonica*, obscuring the equivalent stages across the species. At stage 23, the ungual phalanx is vaguely present in the translucent sheath of *T. scripta* while that of *M. japonica* shows a homogeneous structure in the lateral view. At stage 24, the ungual phalanx is evident in the translucent sheath, but its tip remains unclear in the lateral view in both *T. scripta* and *M. japonica*. The ungual phalanx (including its tip) becomes evident at stage 25 in these species. Nevertheless, the presence of the ungual phalanx (including its tip) is recognized at an earlier stage in *M. reevesii* equivalent to stage 22 in *T. scripta* (Fig. 4l). Thus, the detection of the ungual phalanx in the translucent sheath hardly distinguishes the following stages in *M. reevesii*. Instead, we defined stage 23 in *M. reevesii* by referring to the development of the palmar scales in *T. scripta* at stage 23 when its palmar surface becomes fully covered by small circular scales (Fig. 4n). The translucent sheath begins to recede from the base of the claw toward its distal end during stage 25 until hatching in *T. scripta*. The recession occurs within a few days of stage 23 in *M. reevesii*, and thus, we investigated stage 24 based on the recession of the translucent sheath as the last embryonic stage in *M. reevesii* (Fig. 4r).

The scleral papillae also appear at different times and duration among species. *Mauremys reevesii* present the scleral papillae at stages 18–20, while *T. scripta* and *M. japonica* present those at stage 18 and 19–20, respectively^24,25^. These variations also occur in other turtles in Yntema’s (1968) stages 16–21^23,26^. The scleral papillae induce the development of the scleral ossicles that are present in all birds and turtles^27^. Although controversial, the functional roles of scleral ossicles are presumably adaptive (e.g., to prevent the eyes from physical deformation or visual accommodation)^28^. Therefore, the interspecific variation in the development of scleral papillae may be involved in species-specific visual adaptation.

One of the most evident differences in external morphology between *T. scripta* and *M. reevesii*/*M. japonica* is the carapacial keel that appears as only one ridge in the former species but as three ridges in the latter two. The three keels appear from stage 19 in *M. reevesii*. Although there is only a single keel running at the midline of the vertebral scute in *T. scripta*, its formation begins at stage 19. The three keels also occur in distantly related turtle species, such as *Sternotherus odoratus* (Chelyroidea, Chelyroidae), which also shows primary evidence of keel development at stage 20^29^. Interestingly, this suggests that the embryonic chronology of the carapacial diversity in turtles is conserved, when staged according to Yntema (1968). Nevertheless, embryos of different species at the same Yntema (1968) stage do not necessarily have the same degree of embryonic development. Thus, the development of certain characters should ideally be discussed based on relative timing throughout development, as in Tokita and Kuratani^30^. Further studies on carapace development around stage 19–20 could reveal whether the genetic mechanisms regulating keel development are the same among species and will elucidate the genetic basis underlying carapacial diversity. The present results show that the development of the external morphology of *M. reevesii* is more similar to that of *M. japonica* than that of *T. scripta*, as expected based on their phylogenetic distance^31^.

We then described the gonadal development of *M. reevesii* from stages 16 to 24 using the histology of the gonads. Although TSP generally begins at early stages, when the sexually undifferentiated gonadal ridge is formed, TSP ends at different stages depending on the incubation temperature^8,9^. In *T. scripta*, the sex cords begin to degenerate at stages 18–19 at 31 °C, but develop into seminiferous tubules at stages 20–21 at 26 °C^8^. Structural differentiation arises as the first evidence of gonadal sex differentiation in *T. scripta* and corresponds to the end of TSP^8,32^. According to the chronology of sexual differentiation of gonadal structures, the results suggest that TSP in *M. reevesii* occurs at stage 16 or earlier and lasts until stages 18–19 and 20–21 at FPT and MPT, respectively. The development of gonads in *M. reevesii* followed similar patterns and chronology to that of *T. scripta*.

The development of external morphology in *M. reevesii* proceeds at a higher rate at FPT than at MPT (Fig. 1b, Table 1, Supplementary Table S2) but remains indistinct between the two temperature regimes throughout the studied developmental stages. Interestingly, gonadal sex differentiation is strongly associated with the stages. We calibrated the initial date of the analysis using egg candling and showed that the incubation days from stage 13 provide clues to predict each stage for FPT and MPT (Fig. 1b, Table 1, Supplementary Table S2). While the egg weight seems to change independently of the embryonic stage (Fig. 1a, Supplementary Table S2), the embryo weight increases with embryonic development, at an insignificant rate between the two incubation temperatures (Fig. 1c, Table 1, Supplementary Table S2), suggesting that the embryo weight can also be a parameter to support staging regardless of incubation temperatures (Fig. 1c). These results facilitate the effective sampling of embryos at target stages in *M. reevesii*.

Effects of incubation temperatures on embryo weight have been previously reported in *M. reevesii*^33^, softshelled turtle *Pelodiscus sinensis*^34^, and chicken^35^. Nevertheless, no general trend between incubation temperatures and embryo weight has been observed, at least from these studies, showing that high incubation temperatures resulted in larger and smaller embryo weight in chicken^35^ and *M. reevesii*^33^, respectively, and intermediate incubation temperature resulted in larger embryo weight in *P. sinensis*^34^. Wei et al. (2021) ^33^ showed that the difference in body mass of *M. reevesii* hatchling becomes insignificant by the age of 12 months. Therefore, whether the distinct thermal sensitivities in embryo weight could be explained by any adaptive significance remains elusive. As Yabe^36^ showed that wild-caught individuals of *M. reevesii* in Japan were larger in female than male, factors, such as the growth rate or the longevity, after hatching could have a more important effect on sexual dimorphism in body size.

The present study described the development of external and gonadal morphology in *M. reevesii* under two temperature regimes, namely FPT and MPT, and recorded the time required to reach each stage. The external morphology follows developmental trajectory indistinct between FPT and MPT but the gonadal structures sexually differentiate in temperature-dependent manner after stages 18–19 and 20–21 at FPT and MPT, respectively. We showed that the embryonic stages and associated state of the gonadal differentiation in *M. reevesii* are well predictable as a function of the incubation days and temperatures. Stages at which gonadal structures remain sexually undifferentiated are particularly important as often be the target for *in ovo* experiments on sex determination. Thus, the current study establishes the basis for designing *in ovo* functional analysis of target molecules using pharmacological treatments and/or genetic manipulations via viral infections. Further intra- and interspecific comparisons of TSD species including *M. reevesii* will further explain how temperature stimuli regulate genetic responses towards sex determination.

## Materials and methods

All experiments involving animals and their care were conducted in compliance with ARRIVE guidelines^37,38^. All experiments were performed in accordance with relevant guidelines and regulations. The present study was approved by the Animal Care and Use Committee at the Tokyo University of Science (No. K22011). Turtle eggs were purchased from Kondo farm in Maniwa, Okayama, Japan from June to August 2020 and from June to July 2021. The eggs were collected by farmers 1–3 days from oviposition, but some older eggs may have been accidentally included. The eggs were then transported to the laboratory at Tokyo University of Science, where they were immediately divided into two groups at random and incubated in environmental chambers (Panasonic MIR-554-PJ, Sanyo MIR-253, or Sanyo MIR-153) at a constant temperature of 31 °C (FPT) or 26 °C (MPT). The incubation containers (length × width × height = 23.5 cm × 16.5 cm × 4 cm) were half-filled with vermiculite:water (1:1 ratio) (Setogahara Kaen, Kidori, Gunma, Japan). Each container housed up to 20 uniformly arranged eggs and was placed in a ziplocked plastic bag to maintain humidity. Every three or four days, the containers were ventilated, rehydrated, and rotated in the chamber to reduce the influence of potential thermal heterogeneity. Although the exact date of oviposition was unknown, the appearance of the pigmented eye, observed using the egg candling technique (pigmentation becomes denser with embryonic development), was used to standardize the ages of the eggs. Thus, the initial date at which eye pigmentation was observed was recorded as Day 0 of the investigation.

Embryonic and gonadal development was investigated using 198 embryos of *M. reevesii*. Egg weight was recorded on the day of arrival and of dissection. Five to six embryos were dissected every sampling day for each temperature treatment in phosphate-buffered saline and under a microscope (Olympus SZ61), and then weighed without the extraembryonic membranes. Gonad-mesonephros complex (GMC) of some of embryos were excised for histological analysis on gonadal development. Whole body and GMC were fixed in 4 % paraformaldehyde overnight at 4 °C and then in methanol at increasing concentrations (25 %, 50 %, 75 %, and 100 %). The fixed embryos were photographed with or without the microscope using a camera (Olympus TG-6), examined, and staged referring to the criteria described for *Chelydra serpentina*^23^ and phylogenetically more closely related species, *T. scripta* and *M. japonica*, to *M. reevesii*^24,25,31^. In *T. scripta* and *M. japonica*, stages 13–25 were defined primarily based on forelimb morphology; therefore, forelimb morphology was also used herein as a primary criterion for staging in *M. reevesii*. In the present study, forelimb morphology between adjacent stages was sometimes indistinct (see “Results”). In such cases, other characteristics, such as eye or carapace development, were referenced in combination with forelimb morphology as the primary criterion. For clarity when describing morphological features, the embryos were rotated, and the lateral view was fixed. In other words, for describing stages 13–16, the lateral view is set as the optic fissure is at the bottom of the microscopic field. For describing stages 17–18, the lateral view is set as the line of the upper jaw lies horizontally in the microscopic field.

We investigated the factors that help to predict the embryonic stages by conducting ordinary least squares (OLS) analyses in which the embryonic stage was used as the response variable, while the changes in egg weight, the incubation days, the embryo weight, and the first order interaction between the incubation temperatures and those three variables were used as the explanatory variables. The embryo weight was log-transformed when analyzed. The OLS regression was fitted to the data using the *lm* function in R. Then, we analyzed how well, if any, the variable(s) correlate with the embryonic stage based on the Pearson’s correlation coefficient. Stage 24 in *M. reevesii* lasts until hatching as it is the last and pre-hatching stage. Unlike earlier stages, the duration of stage 24 depends on the timing of hatching rather than changes in specific key morphological characters. Thus, we excluded the parameters at stage 24 from the statistical analyses. All statistical analyses were performed using R (version 4.1.0; https://cran.r-project.org), and graphs were produced using the R package “ggplot2”^39^.

Histological analysis of the gonads was conducted for each of the embryonic stages at both incubation temperatures. The fixed GMC were embedded in histological paraffin and cut into 8-µm thick sections. Each section was stained with hematoxylin and eosin (HE) using a standard procedure and photographed under a microscope (Olympus BX53) with a mounted digital camera (Olympus DP72). Images and graphs were edited and formatted using programs Preview 11.0 (Apple, Inc.), Adobe Photoshop (version 23.1.1), and Adobe Illustrator (version 26.0.2). The description and identification of the gonadal structures followed the terminology proposed for *T. scripta* by Wibbels et al. (1991)^8^.

## Supplementary Information

Supplementary Information.

## Data Availability

Raw pictures in Figs. 2, 3, 4, 5, 6 and in Supplementary Figures S1–S5 are available upon request. Parameters used to produce Fig. 1 are listed in Supplementary Table 1 and R scripts utilized herein are available upon request. The results of the statistical analysis (i.e., the ordinary least square analysis) are summarized in Supplementary Table S2.
