# Supplementary material for: Chronology of embryonic and gonadal development in the Reeves’ turtle, Mauremys reevesii
Source: Sci Rep. 2022 Jul 8;12:11619. doi: 10.1038/s41598-022-15515-w (PMC9270433; doi:10.1038/s41598-022-15515-w)

**Supplementary Information**

**Title: Chronology of embryonic and gonadal development in the Reeves' turtle, *Mauremys reevesii***

Author: Hiroshi Akashi[1]*, Manami Kubota[1], Hibiki Yamamoto[1], Kaori Miyaoku[1,2], Genki Yamagishi[1], Shinichi Miyagawa[1,2]

[1]Department of Biological Science and Technology, Faculty of Advanced Engineering, Tokyo University of Science, 6-3-1 Niijuku, Katsushika-ku, Tokyo 125-8585, Japan
[2]Research Institute for Science and Technology, Tokyo University of Science, Tokyo, Japan, 125-8585, Japan

*Correspondence:
Hiroshi Akashi
Hiroshi-AKASHI@hotmail.co.jp

**Supplementary Table S1** ------------------------------------------------------------------------ 2
Parameters recorded during the embryonic development of
*Mauremys reevesii* at stages 13–24 incubated at female- and
male-producing temperatures (FPT 31 °C and MPT 26 °C, respectively).

**Supplementary Table S2** ------------------------------------------------------------------------ 4
Results of ordinary least squares (OLS) analyses performed on
parameters obtained from the embryonic development of *Mauremys reevesii*.

**Supplementary Figure S1** ------------------------------------------------------------------------ 5
Stages 13–24 of *Mauremys reevesii* in dorsal view.

**Supplementary Figure S2** ------------------------------------------------------------------------ 6
Stages 13–24 of *Mauremys reevesii* in ventral view.

**Supplementary Figure S3** ------------------------------------------------------------------------ 7
The right forelimb of *Mauremys reevesii* at stages 13–24 in dorsal view.

**Supplementary Figure S4** ------------------------------------------------------------------------ 8
The right forelimb of *Mauremys reevesii* at stages 13–24 in ventral view.

**Supplementary Figure S5**. ----------------------------------------------------------------------- 9
The intact gonad-mesonephros complex (GMC) of
*Mauremys reevesii* at stage 24, or pre-hatching stage.

**Supplementary Table S1.** Parameters recorded during the embryonic development of *Mauremys reevesii* at stages 13–24 incubated at female- and male-producing temperatures (FPT 31 °C and MPT 26 °C, respectively).

| Egg_id | Temperature | Initial_egg_weight_g | Egg_weight_at_dissection_g | Egg_delta_weight_g | Embryo_weight_g | Stage | Days |
|---|---|---|---|---|---|---|---|
| 5363 | FPT | 10.1 | 10.06 | -0.04 | 0.04 | 13 | 0 |
| 5517 | FPT | 11.5 | 11.51 | 0.01 | 0.07 | 13 | 0 |
| 5494 | FPT | 11.7 | 11.74 | 0.04 | 0.04 | 13 | 0 |
| 5446 | FPT | 10.8 | 11.06 | 0.26 | 0.04 | 13 | 0 |
| 5392 | FPT | 9.7 | 9.76 | 0.06 | 0.06 | 14 | 0 |
| 5409 | FPT | 14 | 13.92 | -0.08 | 0.05 | 14 | 2 |
| 5457 | FPT | 13.8 | 13.73 | -0.07 | 0.04 | 14 | 2 |
| 5502 | FPT | 12.5 | 12.46 | -0.04 | 0.09 | 14 | 2 |
| 5506 | FPT | 12.1 | 12.1 | 0 | 0.07 | 14 | 2 |
| 5458 | FPT | 11.4 | NA | NA | 0.07 | 14 | 2 |
| 5512 | FPT | 13.1 | 13.04 | -0.06 | 0.11 | 15 | 4 |
| 7955 | FPT | NA | 12.5 | NA | 0.1 | 15 | 4 |
| 5445 | FPT | 12.6 | 12.29 | -0.31 | 0.16 | 16 | 4 |
| 5436 | FPT | 12.3 | 12.26 | -0.04 | 0.12 | 16 | 4 |
| 5481 | FPT | 13.6 | 13.56 | -0.04 | 0.12 | 16 | 4 |
| 5526 | FPT | 12.1 | 12.15 | 0.05 | 0.13 | 16 | 4 |
| 5357 | FPT | 13.8 | 13.79 | -0.01 | 0.17 | 16 | 5 |
| 5365 | FPT | 15.7 | 15.74 | 0.04 | 0.18 | 16 | 5 |
| 7956 | FPT | NA | 11.6 | NA | 0.08 | 16 | 5 |
| 5407 | FPT | 10.5 | 10.49 | -0.01 | 0.18 | 17 | 5 |
| 5432 | FPT | 14.1 | 14.14 | 0.04 | 0.2 | 17 | 5 |
| 5373 | FPT | 11.9 | 11.95 | 0.05 | 0.22 | 17 | 5 |
| 5463 | FPT | 13 | 12.96 | -0.04 | 0.18 | 17 | 6 |
| 5353 | FPT | 11.6 | 11.57 | -0.03 | 0.22 | 17 | 6 |
| 5433 | FPT | 13.1 | 13.07 | -0.03 | 0.23 | 17 | 6 |
| 5441 | FPT | 12.1 | 12.08 | -0.02 | 0.22 | 17 | 6 |
| 5355 | FPT | 11.5 | 11.58 | 0.08 | 0.19 | 17 | 6 |
| 5455 | FPT | 10.4 | 10.37 | -0.03 | 0.25 | 17 | 7 |
| 5344 | FPT | 10.4 | 10.39 | -0.01 | 0.26 | 17 | 7 |
| 5405 | FPT | 11.3 | 11.3 | 0 | 0.23 | 17 | 7 |
| 5486 | FPT | 13.1 | 13.02 | -0.08 | 0.25 | 18 | 7 |
| 5488 | FPT | 12.7 | 12.67 | -0.03 | 0.26 | 18 | 7 |
| 9764 | FPT | NA | 12.2 | NA | 0.25 | 18 | 8 |
| 5417 | FPT | 15.2 | 15.12 | -0.08 | 0.35 | 18 | 9 |
| 5404 | FPT | 12.6 | 12.58 | -0.02 | 0.36 | 18 | 9 |
| 5434 | FPT | 14.2 | 14.23 | 0.03 | 0.35 | 18 | 9 |
| 5342 | FPT | 14.1 | 14.19 | 0.09 | 0.32 | 18 | 9 |
| 9904 | FPT | NA | NA | NA | NA | 18 | 9 |
| 5337 | FPT | 12.7 | 12.72 | 0.02 | 0.45 | 19 | 9 |
| 5412 | FPT | 11.5 | 11.41 | -0.09 | 0.47 | 19 | 10 |
| 5487 | FPT | 13.9 | 13.85 | -0.05 | 0.41 | 19 | 10 |
| 5408 | FPT | 13.1 | 13.09 | -0.01 | 0.46 | 19 | 10 |
| 5396 | FPT | 12.8 | 12.81 | 0.01 | 0.41 | 19 | 10 |
| 5489 | FPT | 13.1 | 13.11 | 0.01 | 0.41 | 19 | 10 |
| 5443 | FPT | 13.2 | 13.2 | 0 | 0.49 | 19 | 11 |
| 5406 | FPT | 11.8 | 11.89 | 0.09 | 0.4 | 19 | 11 |
| 5338 | FPT | 10.2 | 10.44 | 0.24 | 0.41 | 19 | 11 |
| 5352 | FPT | 13.9 | 13.88 | -0.02 | 0.48 | 20 | 11 |
| 5440 | FPT | 11.1 | 11.52 | 0.42 | 0.85 | 20 | 11 |
| 8336 | FPT | NA | 12.49 | NA | 0.6 | 20 | 12 |
| 9865 | FPT | NA | 14 | NA | 0.6 | 20 | 12 |
| 8337 | FPT | NA | 11.21 | NA | 0.59 | 20 | 13 |
| 5429 | FPT | 14.8 | 15 | 0.2 | 0.61 | 21 | 14 |
| 5426 | FPT | 13 | 13.22 | 0.22 | 1.1 | 21 | 14 |
| 9739 | FPT | NA | NA | NA | 1.1 | 21 | 15 |
| 9867 | FPT | NA | NA | NA | NA | 21 | 15 |
| 9749 | FPT | NA | NA | NA | 1.1 | 21 | 16 |
| 9750 | FPT | NA | NA | NA | 1.1 | 21 | 16 |
| 8338 | FPT | NA | 11.23 | NA | 0.58 | 21 | NA |
| 8339 | FPT | NA | 10 | NA | 0.55 | 21 | NA |
| 5527 | FPT | 12.4 | 13 | 0.6 | 1.02 | 22 | 14 |
| 5411 | FPT | 11 | 10.91 | -0.09 | 1.37 | 22 | 14 |
| 5389 | FPT | 11 | 10.92 | -0.08 | 0.82 | 22 | 14 |
| 9741 | FPT | NA | NA | NA | 1 | 22 | 15 |
| 5383 | FPT | 12.6 | 12.4 | -0.2 | 1.14 | 22 | 17 |
| 5345 | FPT | 13.2 | 13.11 | -0.09 | 1.09 | 22 | 17 |
| 5476 | FPT | 10.8 | 10.82 | 0.02 | 1.14 | 22 | 17 |
| 5349 | FPT | 12.1 | 12.16 | 0.06 | 1.36 | 22 | 17 |
| 5414 | FPT | 12.3 | 12.44 | 0.14 | 1.23 | 22 | 17 |
| 5427 | FPT | 12.9 | NA | NA | NA | 22 | NA |
| 9754 | FPT | NA | NA | NA | 2.1 | 23 | 19 |
| 9755 | FPT | NA | NA | NA | 1.8 | 23 | 19 |
| 9756 | FPT | NA | NA | NA | 1.9 | 23 | 19 |
| 5424 | FPT | 13.5 | 13.86 | 0.36 | 1.74 | 23 | 20 |
| 5449 | FPT | 14.1 | 14.75 | 0.65 | 1.9 | 23 | 20 |
| 5478 | FPT | 12.2 | 13.51 | 1.31 | 2.05 | 23 | 20 |
| 5415 | FPT | 12.3 | 12.79 | 0.49 | 2.46 | 24 | 20 |
| 5508 | FPT | 14.8 | 15.49 | 0.69 | 2.32 | 24 | 20 |
| 5438 | FPT | 13 | 13.09 | 0.09 | 3.05 | 24 | 23 |
| 5399 | FPT | 12.5 | 12.68 | 0.18 | 2.7 | 24 | 23 |
| 5364 | FPT | 10.1 | 10.38 | 0.28 | 2.75 | 24 | 23 |
| 5362 | FPT | 11.9 | 12.32 | 0.42 | 2.49 | 24 | 23 |
| 5410 | FPT | 12.2 | 12.8 | 0.6 | 2.65 | 24 | 23 |
| 5439 | FPT | 12.7 | 12.92 | 0.22 | 4.34 | 24 | 26 |
| 5360 | FPT | 12.1 | 12.49 | 0.39 | 3.17 | 24 | 26 |
| 5477 | FPT | 14.5 | 15.05 | 0.55 | 3.61 | 24 | 26 |
| 5377 | FPT | 12.7 | 13.33 | 0.63 | 4.54 | 24 | 26 |
| 5454 | FPT | 11.5 | 12.55 | 1.05 | 3.38 | 24 | 26 |
| 5366 | FPT | 11 | 10.95 | -0.05 | 5.1 | 24 | 32 |
| 5480 | FPT | 12.3 | 12.57 | 0.27 | 5.57 | 24 | 32 |
| 5375 | FPT | 14 | 14.35 | 0.35 | 5.78 | 24 | 32 |
| 5529 | FPT | 12.6 | 13.25 | 0.65 | 4.74 | 24 | 32 |
| 5343 | FPT | 13.8 | NA | NA | 4.3 | 24 | 32 |
| 5504 | FPT | 12 | 12.32 | 0.32 | 6.4 | 24 | 38 |
| 5503 | FPT | 12.6 | 13.01 | 0.41 | 6.18 | 24 | 38 |
| 5379 | FPT | 12.1 | 12.72 | 0.62 | 6.6 | 24 | 38 |
| 5498 | FPT | 12.2 | 12.93 | 0.73 | 5.8 | 24 | 38 |
| 5420 | FPT | 10.8 | 11.65 | 0.85 | 6.05 | 24 | 38 |
| 5496 | FPT | 15.8 | 16.14 | 0.34 | 8.19 | 24 | 46 |
| 5462 | FPT | 14.9 | 16.16 | 1.26 | 7.19 | 24 | 46 |
| 5350 | FPT | 13.3 | NA | NA | 5.87 | 24 | 46 |

**Supplementary Table S1.** Continued.

| Egg_id | Temperature | Initial_egg_weight_g | Egg_weight_at_dissection_g | Egg_delta_weight_g | Embryo_weight_g | Stage | Days |
|---|---|---|---|---|---|---|---|
| 5280 | MPT | 12.32 | 12.09 | -0.23 | 0.04 | 13 | 0 |
| 5328 | MPT | 11.25 | 11.22 | -0.03 | NA | 13 | 0 |
| 5299 | MPT | 12.86 | 12.86 | 0 | NA | 13 | 0 |
| 5315 | MPT | 12.77 | 12.82 | 0.05 | 0.01 | 13 | 0 |
| 5151 | MPT | 12.46 | 12.41 | -0.05 | 0.05 | 14 | 3 |
| 5186 | MPT | 14.71 | 14.66 | -0.05 | 0.05 | 14 | 3 |
| 5138 | MPT | 10.92 | 10.9 | -0.02 | 0.06 | 14 | 3 |
| 5148 | MPT | 14.43 | 14.41 | -0.02 | 0.06 | 14 | 3 |
| 5226 | MPT | 12.37 | 12.29 | -0.08 | 0.1 | 14 | 6 |
| 5228 | MPT | 12.07 | 12 | -0.07 | 0.08 | 14 | 6 |
| 5214 | MPT | 13.44 | 13.41 | -0.03 | 0.13 | 14 | 6 |
| 5218 | MPT | 14.21 | 14.2 | -0.01 | 0.09 | 14 | 6 |
| 5178 | MPT | 12.98 | 12.98 | 0 | 0.13 | 15 | 8 |
| 5217 | MPT | 14.57 | 14.57 | 0 | 0.11 | 15 | 8 |
| 7230 | MPT | NA | 9.9 | NA | 0.12 | 15 | 8 |
| 7231 | MPT | NA | 9.96 | NA | 0.11 | 15 | 8 |
| 5249 | MPT | 13.33 | 13.3 | -0.03 | 0.18 | 16 | 8 |
| 7224 | MPT | NA | 12.94 | NA | 0.15 | 16 | 8 |
| 5269 | MPT | 12.26 | 12.22 | -0.04 | 0.17 | 16 | 10 |
| 5282 | MPT | 12.6 | 12.56 | -0.04 | 0.16 | 16 | 10 |
| 5240 | MPT | 11.89 | 11.87 | -0.02 | 0.18 | 16 | 10 |
| 5291 | MPT | 13.8 | 13.81 | 0.01 | 0.18 | 16 | 10 |
| 7226 | MPT | NA | 12.64 | NA | 0.26 | 17 | 11 |
| 7227 | MPT | NA | 13.21 | NA | 0.23 | 17 | 11 |
| 5335 | MPT | 12.4 | 12.39 | -0.01 | 0.23 | 17 | 12 |
| 5322 | MPT | 10.98 | 11 | 0.02 | 0.25 | 17 | 12 |
| 5303 | MPT | 12.05 | 12.09 | 0.04 | 0.25 | 17 | 12 |
| 5320 | MPT | 8.11 | 8.45 | 0.34 | 0.24 | 17 | 12 |
| 5142 | MPT | 13.36 | 13.32 | -0.04 | 0.28 | 17 | 14 |
| 7228 | MPT | NA | 10.43 | NA | 0.29 | 18 | 13 |
| 5183 | MPT | 12.24 | 12.14 | -0.1 | 0.32 | 18 | 14 |
| 5173 | MPT | 15.1 | 15.05 | -0.05 | 0.33 | 18 | 14 |
| 5167 | MPT | 13.65 | 13.61 | -0.04 | 0.32 | 18 | 14 |
| 7229 | MPT | NA | 10.65 | NA | 0.35 | 18 | 15 |
| 9797 | MPT | NA | 11.2 | NA | 0.29 | 18 | 15 |
| 5222 | MPT | 14.72 | 14.7 | -0.02 | 0.4 | 18 | 16 |
| 5187 | MPT | 11.39 | 11.39 | 0 | 0.32 | 18 | 16 |
| 7233 | MPT | NA | 11.63 | NA | 0.37 | 18 | 17 |
| 7234 | MPT | NA | 11.28 | NA | 0.38 | 18 | 17 |
| 5275 | MPT | 14 | 13.99 | -0.01 | 0.41 | 18 | 18 |
| 7225 | MPT | NA | 14.04 | NA | 0.33 | 18 | NA |
| 7960 | MPT | NA | 14.78 | NA | 0.31 | 18 | NA |
| 5262 | MPT | 10.7 | 11.11 | 0.41 | 0.56 | 19 | 16 |
| 9798 | MPT | NA | 14 | NA | 0.5 | 19 | 17 |
| 9799 | MPT | NA | 14.3 | NA | 0.51 | 19 | 17 |
| 5242 | MPT | 13.48 | 13.49 | 0.01 | 0.51 | 19 | 18 |
| 5301 | MPT | 7.72 | 7.77 | 0.05 | 0.57 | 19 | 18 |
| 5293 | MPT | 10.72 | 10.79 | 0.07 | 0.53 | 19 | 18 |
| 5313 | MPT | 12.56 | 12.53 | -0.03 | 0.53 | 20 | 20 |
| 5327 | MPT | 12.39 | 12.37 | -0.02 | 0.49 | 20 | 20 |
| 5332 | MPT | 11.29 | 11.27 | -0.02 | 0.58 | 20 | 20 |
| 5307 | MPT | 11.55 | 11.57 | 0.02 | 0.53 | 20 | 20 |
| 5256 | MPT | 13.02 | 13 | -0.02 | 0.85 | 20 | 22 |
| 5143 | MPT | 11.21 | 11.23 | 0.02 | 0.86 | 21 | 22 |
| 5159 | MPT | 14.84 | 15.4 | 0.56 | 0.99 | 21 | 22 |
| 5145 | MPT | 12.5 | 13.9 | 1.4 | 0.73 | 21 | 22 |
| 5231 | MPT | 13.57 | 13.52 | -0.05 | 0.99 | 21 | 24 |
| 5169 | MPT | 13.18 | 13.15 | -0.03 | 0.99 | 21 | 24 |
| 5139 | MPT | 11.98 | 12 | 0.02 | 1.05 | 21 | 24 |
| 9805 | MPT | NA | 12.6 | NA | 1.1 | 21 | 24 |
| 9801 | MPT | NA | NA | NA | 1.3 | 21 | 25 |
| 9811 | MPT | NA | 13.7 | NA | 1.6 | 21 | 29 |
| 5152 | MPT | 13.84 | 13.8 | -0.04 | 1.2 | 22 | 24 |
| 9802 | MPT | NA | 14 | NA | 1.3 | 22 | 25 |
| 9809 | MPT | NA | 13.5 | NA | 1.6 | 22 | 25 |
| 5156 | MPT | 13.61 | 13.66 | 0.05 | 1.44 | 22 | 26 |
| 5150 | MPT | 13.51 | 13.47 | -0.04 | 1.55 | 22 | 27 |
| 5149 | MPT | 14.85 | 14.81 | -0.04 | 1.22 | 22 | 27 |
| 5147 | MPT | 11.22 | 11.19 | -0.03 | 1.32 | 22 | 27 |
| 5185 | MPT | 10.84 | 10.98 | 0.14 | 1.76 | 22 | 30 |
| 9815 | MPT | NA | 12 | NA | 2 | 23 | 27 |
| 9817 | MPT | NA | 12 | NA | 1.5 | 23 | 27 |
| 9814 | MPT | NA | NA | NA | 2.3 | 23 | 28 |
| 9812 | MPT | NA | NA | NA | 2.1 | 23 | 29 |
| 5179 | MPT | 13.28 | 13.24 | -0.04 | 1.97 | 23 | 30 |
| 5140 | MPT | 12.66 | 12.66 | 0 | 1.82 | 23 | 30 |
| 5144 | MPT | 15.07 | 15.31 | 0.24 | 2.22 | 23 | 30 |
| 5181 | MPT | 10.56 | 10.45 | -0.11 | 2.06 | 23 | 33 |
| 5172 | MPT | 12.68 | 12.69 | 0.01 | 2.6 | 24 | 33 |
| 5153 | MPT | 11.79 | 12.27 | 0.48 | 2.68 | 24 | 33 |
| 5194 | MPT | 14.55 | 14.56 | 0.01 | 3.23 | 24 | 36 |
| 5264 | MPT | 12.08 | 12.31 | 0.23 | 3.34 | 24 | 36 |
| 5182 | MPT | 12.02 | 12.58 | 0.56 | 4.02 | 24 | 36 |
| 5260 | MPT | 15.71 | 16.51 | 0.8 | 4.71 | 24 | 36 |
| 5216 | MPT | 14.22 | 14.42 | 0.2 | 5.06 | 24 | 40 |
| 5235 | MPT | 15.09 | 15.4 | 0.31 | 4.72 | 24 | 40 |
| 5220 | MPT | 10.75 | 11.08 | 0.33 | 3.96 | 24 | 40 |
| 5207 | MPT | 13.5 | 14.08 | 0.58 | 4.58 | 24 | 40 |
| 5192 | MPT | 13.77 | 13.9 | 0.13 | 6.02 | 24 | 44 |
| 5224 | MPT | 12.24 | 12.44 | 0.2 | 5.67 | 24 | 44 |
| 5219 | MPT | 13.65 | 14 | 0.35 | 6.33 | 24 | 44 |
| 5193 | MPT | 13.73 | 14.11 | 0.38 | 6.25 | 24 | 44 |
| 5244 | MPT | 13.57 | 13.72 | 0.15 | 5.81 | 24 | 48 |
| 5255 | MPT | 12.53 | 13.05 | 0.52 | 6.46 | 24 | 48 |
| 5236 | MPT | 12.88 | 13.58 | 0.7 | 6.38 | 24 | 48 |
| 5310 | MPT | 12.58 | 13.55 | 0.97 | 6.65 | 24 | 48 |
| 5190 | MPT | 10.66 | NA | NA | NA | 24 | NA |

**Supplementary Table S2.** Results of ordinary least squares (OLS) analyses performed on parameters obtained from the embryonic development of *Mauremys reevesii* . The parameters include the embryonic stages (i.e., stage), changes in egg weight from the time of arrival to that of dissection (i.e., egg), days required to reach each stage (i.e., day), embryo weight at

| Model | | | Estimate ± SE | *P* -value |
|---|---|---|---|---|
| stage ~ egg*Temp | Intercept | | 17.9 ± 0.4 | < 2.0 × 10^-16 |
| | Slope | egg | 3.3 ± 1.76 | 0.06 |
| | | Temp | -0.23 ± 0.56 | 0.68 |
| | | egg*Temp | 1.64 ± 2.4 | 0.5 |
| | | | | adjusted R-squared: 0.08 |
| stage ~ day*Temp | Intercept | | 12.8 ± 0.13 | < 2.0 × 10^-16 |
| | Slope | **day** | **0.35 ± 0.01** | **< 2.0 × 10^-16** |
| | | **Temp** | **0.89 ± 0.18** | **1.99 × 10^-6** |
| | | **day*Temp** | **0.16 ± 0.01** | **< 2.0 × 10^-16** |
| | | | | adjusted R-squared: 0.97 |
| stage ~ embryo*Temp | Intercept | | 15.7 ± 0.23 | < 2.0 × 10^-16 |
| | Slope | **embryo** | **4.16 ± 0.25** | **< 2.0 × 10^-16** |
| | | Temp | 0.04 ± 0.33 | 0.90 |
| | | embryo*Temp | 0.54 ± 0.39 | 0.17 |
| | | | | adjusted R-squared: 0.78 |

*Slope(s) with significant correlations are shown in boldface.*

**Supplementary Figure S1.** Stages 13–24 of *Mauremys reevesii* in dorsal view. (**a**) Stage 13. (**b**) Stage 14. (**c**) Stage 15. (**d**) Stage 16. (**e**) Stage 17. (**f**) Stage 18. (**g**) Stage 19. (**h**) Stage 20. (**i**) Stage 21. (**j**) Stage 22. (**k**) Stage 23. (**l**) Stage 24. The scale bar is 5 mm.

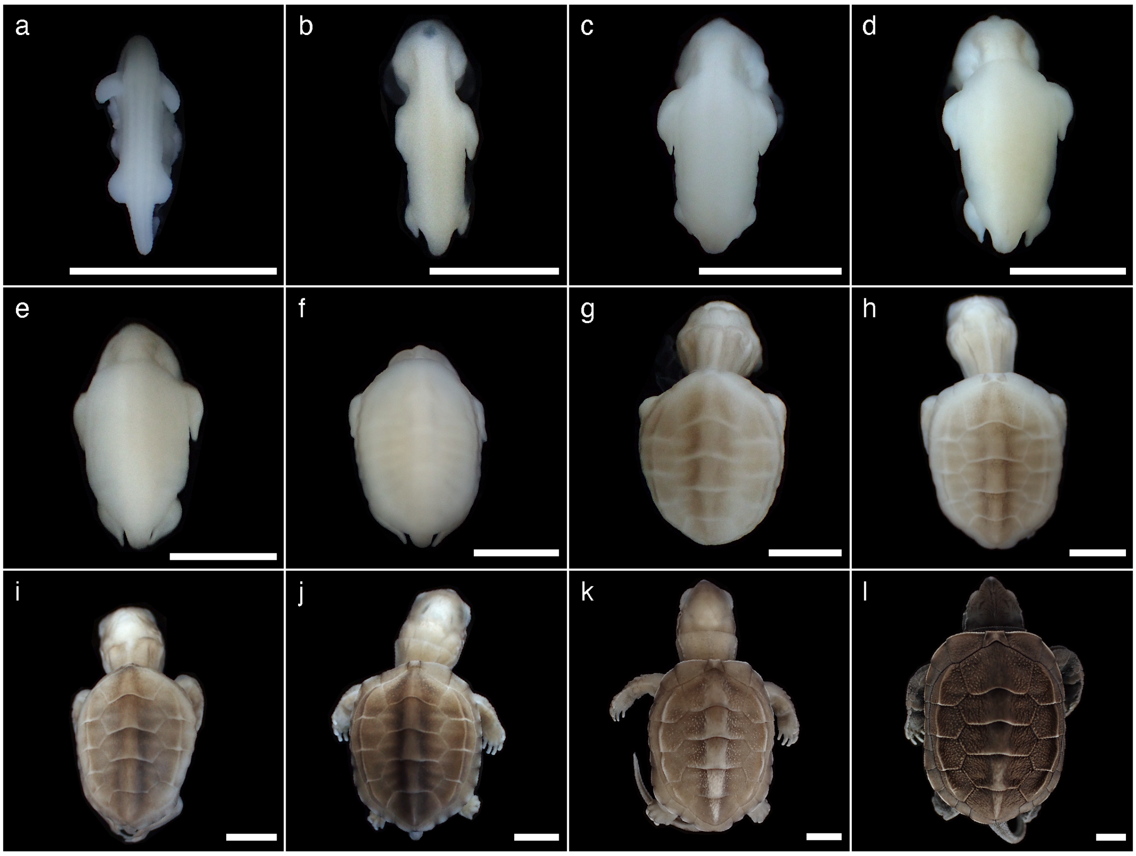

**Supplementary Figure S2.** Stages 13–24 of *Mauremys reevesii* in ventral view. (**a**) Stage 13. (**b**) Stage 14. (**c**) Stage 15. (**d**) Stage 16. (**e**) Stage 17. (**f**) Stage 18. (**g**) Stage 19. (**h**) Stage 20. (**i**) Stage 21. (**j**) Stage 22. (**k**) Stage 23. (**l**) Stage 24. The scale bar is 5 mm.

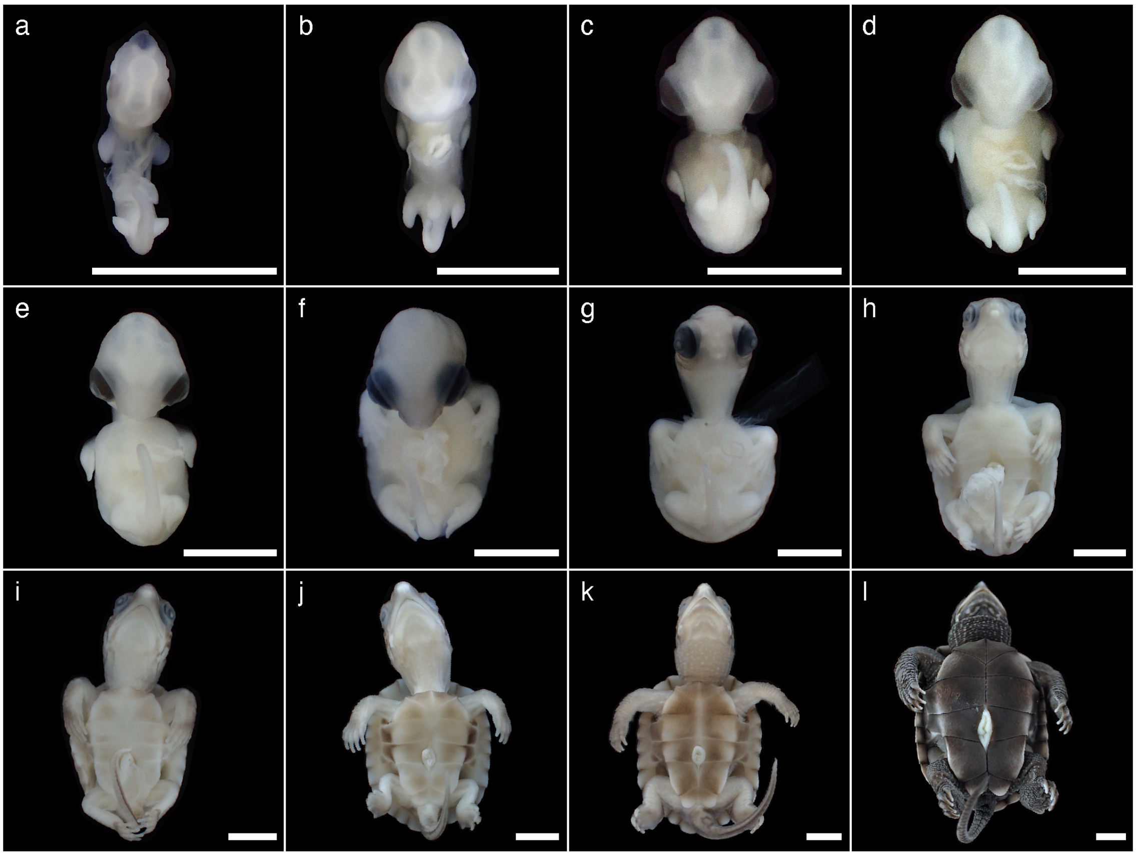

**Supplementary Figure S3**. The right forelimb of *Mauremys reevesii* at stages 13–24 in dorsal view. (**a**) Stage 13. (**b**) Stage 14. (**c**) Stage 15. (**d**) Stage 16. (**e**) Stage 17. (**f**) Stage 18. (**g**) Stage 19. (**h**) Stage 20. (**i**) Stage 21. (**j**) Stage 22. (**k**) Stage 23. (**l**) Stage 24. The scale bar is 1 mm.

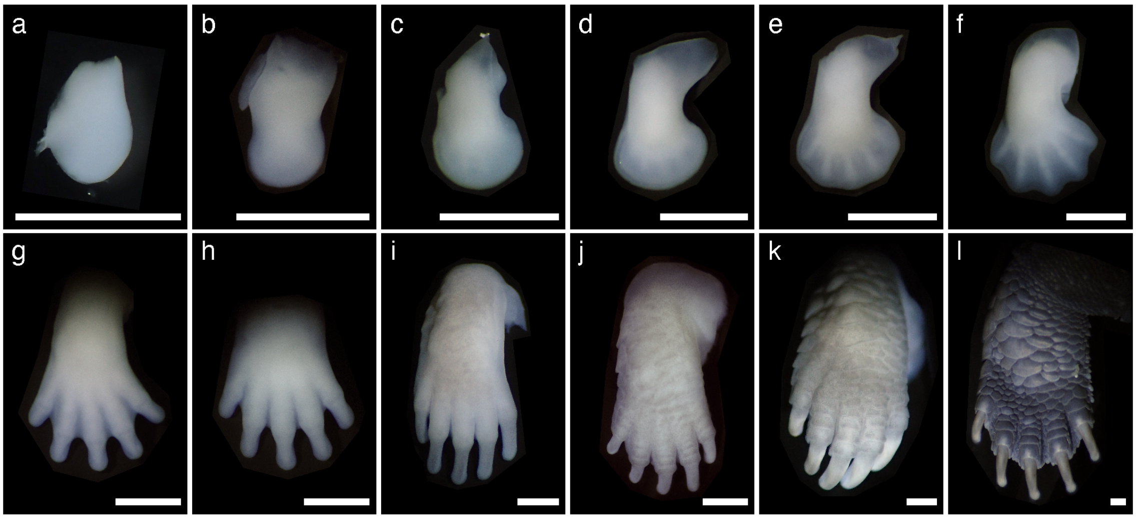

**Supplementary Figure S4**. The right forelimb of *Mauremys reevesii* at stages 13–24 in ventral view. (**a**) Stage 13. (**b**) Stage 14. (**c**) Stage 15. (**d**) Stage 16. (**e**) Stage 17. (**f**) Stage 18. (**g**) Stage 19. (**h**) Stage 20. (**i**) Stage 21. (**j**) Stage 22. (**k**) Stage 23. (**l**) Stage 24. The scale bar is 1 mm.

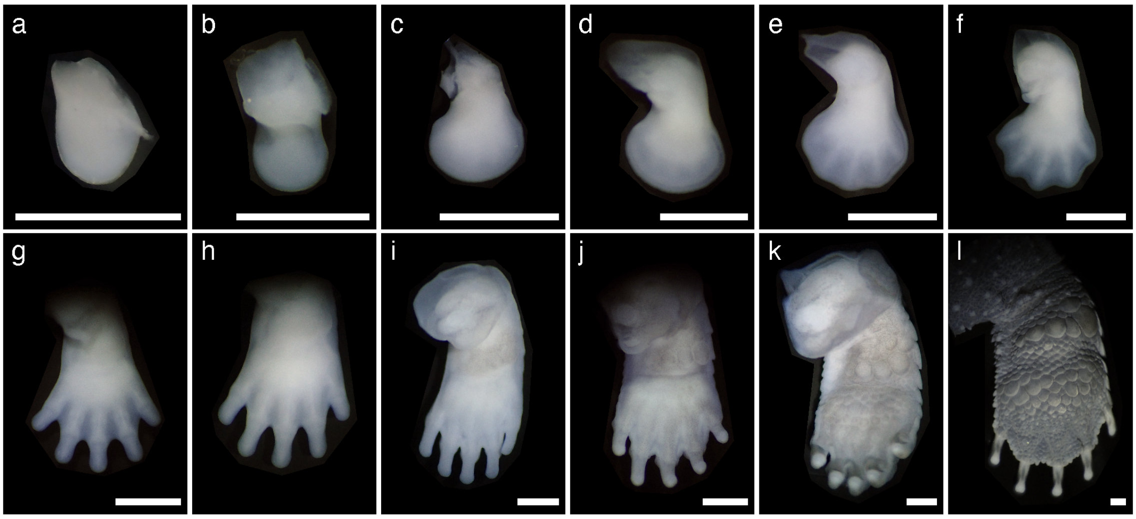

**Supplementary Figure S5**. The intact gonad-mesonephros complex (GMC) of *Mauremys reevesii* at stage 24, or pre-hatching stage. (a) GMC of an embryo incubated at female-producing temperature (31 °C) is shown in ventral view. (b) GMC of an embryo incubated at male-producing temperature (26 °C) is shown in ventral view. Blue dotted line indicates the gonad on left for clarity. The scale bar is 5 mm.

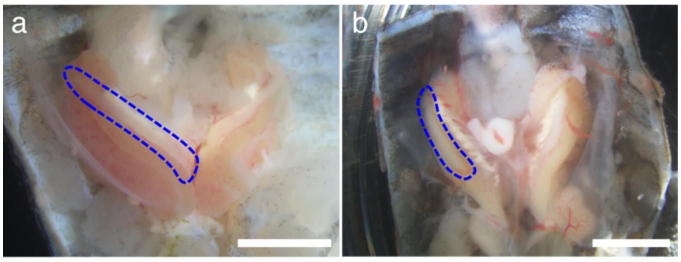

Supplement: Supplementary file 1 — Supplementary Information. [file 41598_2022_15515_MOESM1_ESM.pdf]
